# Supplementary figures and images for: Case-control meta-analysis of blood DNA methylation and autism spectrum disorder
Source: Mol Autism. 2018 Jun 28;9:40. doi: 10.1186/s13229-018-0224-6 (PMC6022498; doi:10.1186/s13229-018-0224-6)

**A**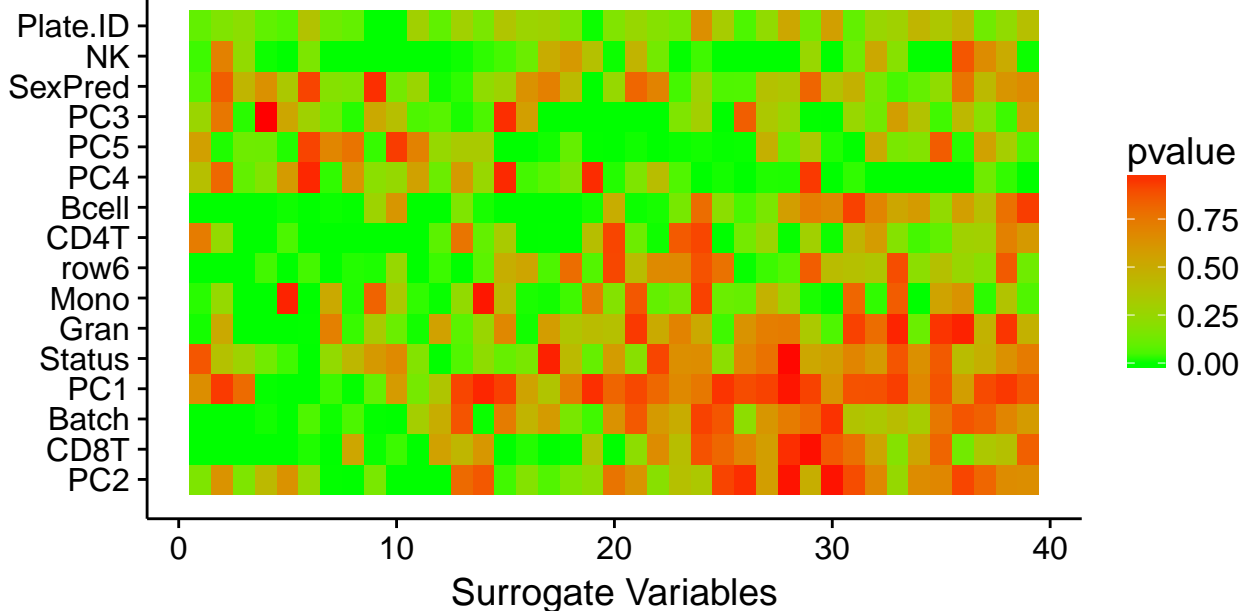**B**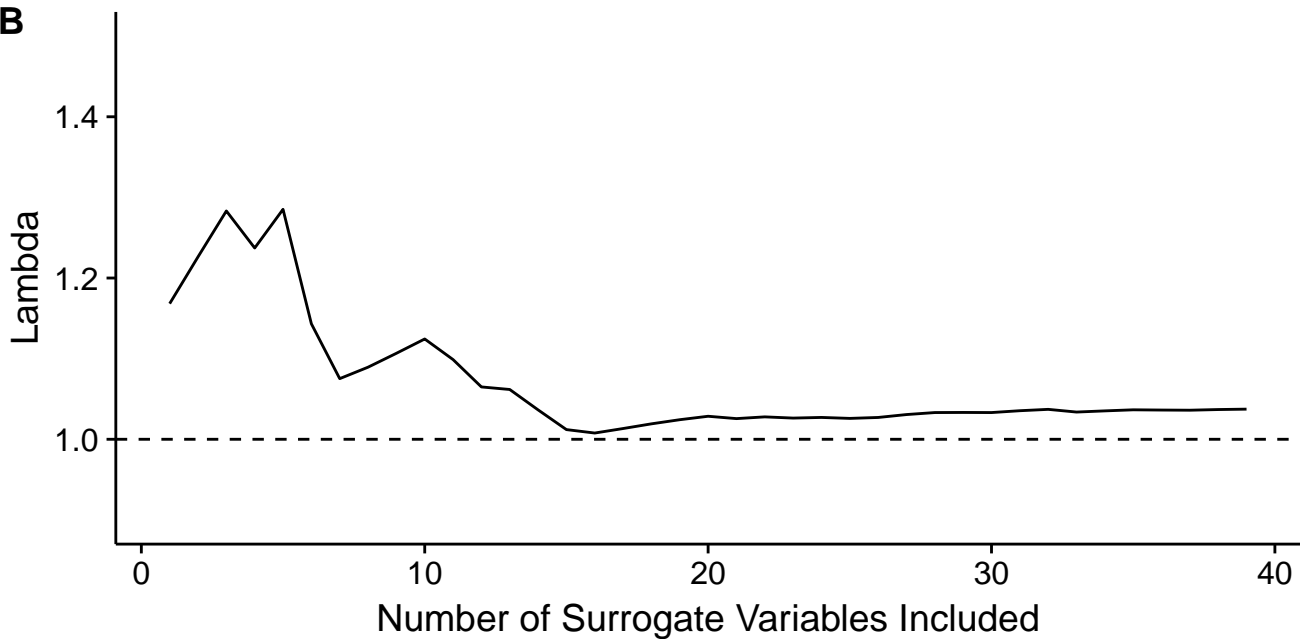

# Variables

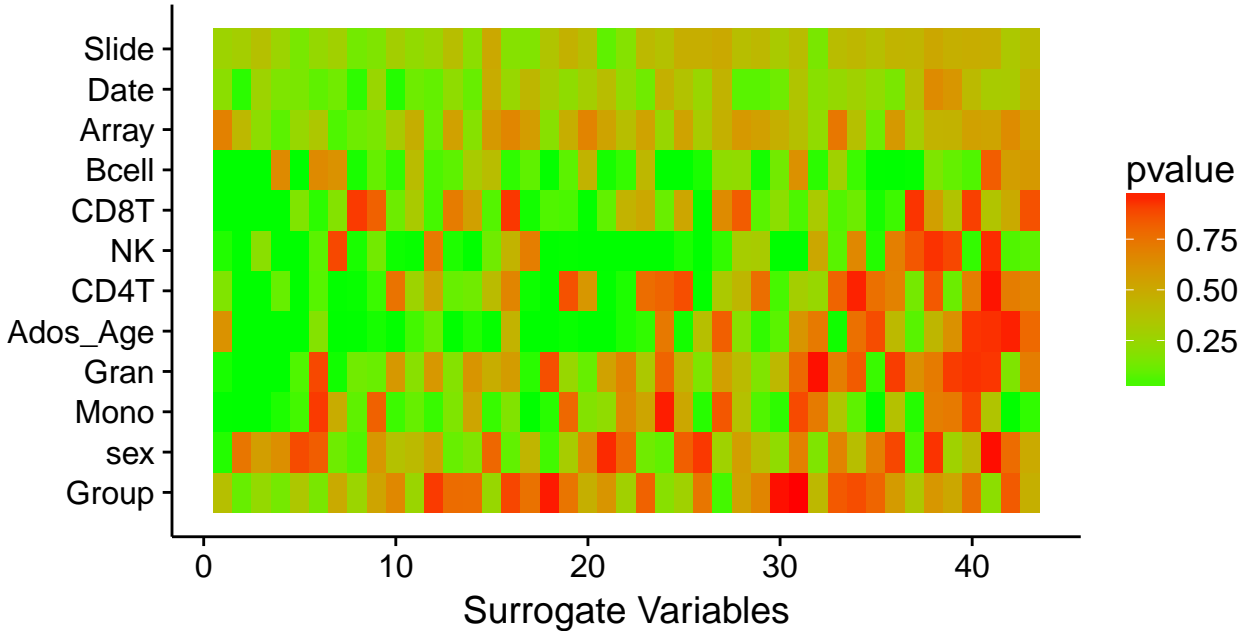

# Lambda

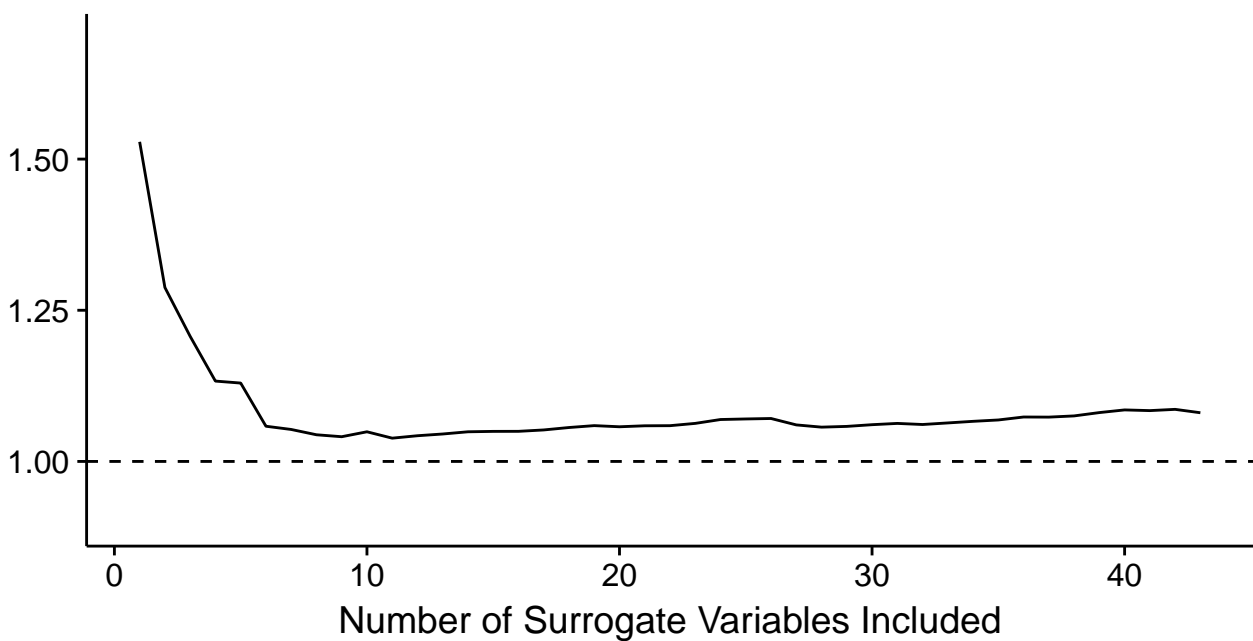

Supplement: Supplementary file 1 — Figures S1-S2. Depiction of surrogate variable selection process for SEED (S1) and SSC (S2). Panel A: Heatmap indicating degree of association with known potential technical variables or confounders with estimated surrogate variables. Panel B: Inflation factor (lambda) calculated for progressively including surrogate variables in association models. The number of surrogate variables to include in the ultimate association testing model was to determine to be that which properly controlled the inflation factor and adequately captured known technical variables or confounders. See “Methods” for additional explanation. (PDF 19 kb) [file 13229_2018_224_MOESM1_ESM.pdf]

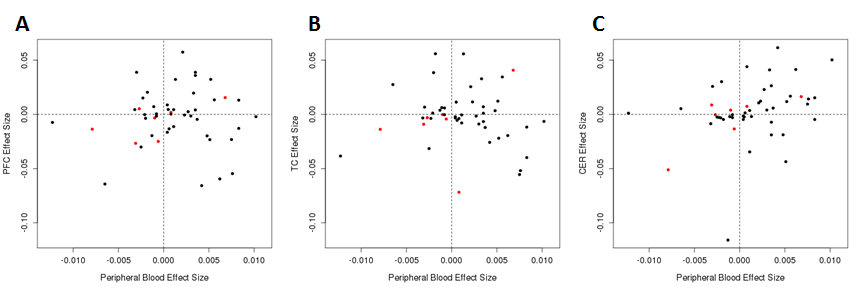

Supplement: Supplementary file 5 — Figures S3. Quadrant plots depicting concordance in effect sizes between suggestively associated (p < 1 × 10− 4) CpG sites in peripheral blood and three brain regions. A) Prefrontal cortex B) Temporal Cortex C) Cerebellum. Points in red indicate those sites with p < 1 × 10− 5 in peripheral blood. (PNG 21 kb) [file 13229_2018_224_MOESM5_ESM.png]
